# Toward a more ‘sovereign’ Europe? Domestic, bilateral, and European factors to explain France’s (growing) influence on EU politics, 2017–2022

**DOI:** 10.1057/s41253-022-00203-y

**Published:** 2023-01-30

**Authors:** Salih Isik Bora, Lucas Schramm

**Affiliations:** 1 Sciences Po Paris, Paris, France; 2 Ludwig-Maximilians-University Munich, Munich, Germany

**Keywords:** France, EU, Influence, Macron, Multi-level, Sovereignty

## Abstract

In recent years, both inside and outside France, scholars and policymakers have emphasized a small and declining French influence on European politics and the political direction of the European Union (EU). By contrast, in 2022, at the end of President Emmanuel Macron’s first term in office, the EU increasingly follows French preferences and ideas. We argue that this renewed French clout is due to the interplay of factors located at different levels of government: a centralized political system and careful preparation of policy objectives at the domestic level, together with a more balanced bilateral relationship with Germany and several exogenous shocks hitting the EU, enabled the French President to upload national policy priorities to the European level. We combine a longer-term perspective, which considers the formulation and pursuit of national strategies, with moments of crisis altering the EU’s status quo and leading member states to promote change. We demonstrate France’s influence on EU politics based on developments in three policy fields, namely fiscal policy, competition policy, and defense industrial policy.

## Introduction

On 26 September 2017, France’s newly elected President, Emmanuel Macron, diagnosed the state of the European Union (EU). Speaking to an audience of students and policymakers at the Sorbonne University in Paris, he outlined a range of ambitious projects for how to reform and advance the EU. He called for a more “sovereign” Europe with a greater capacity to act both internally and in the world (Macron 2017). Among other things, Macron suggested higher EU spending and a strategic culture in the fields of security and defense to ensure “Europe’s autonomous operating capabilities” and reduce its dependence on the United States; a genuine European industrial policy to support the ecological and digital transitions and promote the emergence of “European champions”; and a strong budget, preferably inside Europe’s Economic and Monetary Union (Eurozone), to “fund common expenditure” and provide stability in the event of economic crises.

Deliberately, Macron gave his Sorbonne speech only two days after parliamentary elections had taken place in Germany. Calling on the new German government to engage with his proposals, he put pressure on Chancellor Angela Merkel, who had emerged victorious from the elections and who Macron said was sharing his European commitment, to take a stance on the various EU topics raised. Macron’s Sorbonne speech signaled a renewed French confidence and determination to promote policy initiatives, also with respect to its most important ally in Europe. French-German bilateralism provides both countries with opportunities to shape EU politics (Krotz and Schild 2013). However, caught in difficult and lengthy coalition-building talks, the German government never gave an explicit response to Macron’s speech.

Moreover, by early 2019, Northern EU member states had watered down his proposal for a Eurozone budget to a “Budgetary Instrument for Convergence and Competitiveness” (BICC) amounting to a mere €30 billion. Macron’s idea for a “Buy European Act” in public procurement was quickly dismissed by European Commission officials. As for the proposal for a “common strategic culture,” it was met with little enthusiasm. Such developments seemed to vindicate scholars who emphasize a relatively small French influence on EU politics and an asymmetry in its relationship with Germany (Bulmer and Paterson 2019: 1–20; Webber 2019: 40–55). More broadly, Macron’s discourse on “European sovereignty” seemed little more than the latest refrain of long-standing French ideas about Europe acting as a “shield against globalization.” Scholars of France repeatedly remarked that these ideas had long lost their convincingness in EU politics (Schmidt 2007: 1002). In the domestic political arena, Macron’s contenders criticized the futility of the President’s proposals in a supposedly economically (neo-)liberal and reform-resistant Europe (Paris Match 2017).

And yet, in 2022, by the end of President Macron’s first term in office, themes evoked in the Sorbonne speech have gained considerable resonance. The idea of “European sovereignty” made its way into the political guidelines of the European Commission and, more recently, into European Council Conclusions (Von der Leyen 2019; European Council 2022). Furthermore, as we will examine in this article, such ideas, which Macron and the French government linked to substantive preferences in terms of fiscal policy, competition policy, and defense industrial policy, have materialized. The substantive proposals, respectively, were fiscal mutualization, the reform of state aid and merger control rules, and the European Defence Fund (EDF).

How can one explain these French-inspired European shifts in policies and priorities across different policy fields? And why did they unfold within a remarkably short period of time? We argue that the increased French influence—defined as the power to lead other actors to support the attainment of one’s preferences—is the result of several explanatory factors, located at different levels of government, coming together: first, on the domestic level, the centralized French political system, a solid majority in parliament, and a mandate for EU reform gave President Macron a large room for maneuver. Second, his pro-European rhetoric and appeal to Germany’s European commitment enabled Macron to mobilize and use French-German bilateralism. Third, several exogenous shocks threatening the European polity made the status quo untenable and favored France’s reform-oriented political agenda.

Our framework thus combines long-term and relatively stable factors, like France’s presidential system and traditional European priorities, with short-term and more volatile factors, such as (changes in) member-state relations and moment of deep crisis for the EU. Bringing together elements of structure and agency, we demonstrate conditions and pathways for a (large) member state to significantly alter European politics. In doing so, we also show that France during the Macron Presidency was able to shape the EU’s political direction and outlook to an extent that previous French governments had not managed, and that most scholarly literature would not have expected.

To be sure, we do not claim that France has been influential in *all* EU policy fields to similar extents. Moreover, we do not equal President Macron’s political agency with ‘France’ or the fairly consistent French European preferences. Senior civil servants stress that most policy positions defended by Macron were not new but were rather inherited from previous French governments. For instance, the idea of “European sovereignty” featured prominently in presidential speeches over the past decades (Chirac 2007; Sarkozy 2011; Hollande 2015). However, we do argue and demonstrate that French policy objectives fully came to the fore and made a substantial impact under the Macron Presidency because of the outlined interaction of domestic, bilateral, and European-level factors.

The article is structured as follows. The next section describes the analytical framework, highlighting in greater detail the three levels of analysis and the corresponding explanatory factors. The following section presents the case selection and documents the sources and data used in this study. The empirical analysis traces developments in three EU policy fields, namely fiscal policy, industrial and competition policy, and security and defense. Together, the case studies show how and why French European proposals increasingly gained ground and made a profound and presumably lasting impact on EU politics. The concluding section summarizes the empirical and theoretical implications of the study and puts them into the context of recent political events, such as the French parliamentary elections of June 2022 and Russia’s war against Ukraine.

### Long-term preparation, strategies, and the favorable role of EU crises

To assess and explain French influence on EU politics, this study considers conditions and explanatory factors located at three levels of government, namely the domestic, bilateral, and European level. There are several reasons to take such an approach: first, domestic politics and European policymaking have become increasingly intertwined as the EU gained new competences in policy fields which traditionally had been at the core of the nation state, such as fiscal and defense policy (Genschel and Jachtenfuchs 2013; Hooghe and Marks 2009). More than in the past, EU studies today closely consider developments in the domestic arena when assessing decisions made at the EU level, and vice versa. Research has shown that domestic institutional and administrative structures, together with the political leader’s performance, contribute to determine a country’s influence at the European level. While rather structural elements like the political system and parliamentary majorities function as abilities (or constraints) for influence-making, individual leaders, via their political rhetoric, can push other actors to re-consider their preferences (Lovato 2022).

Second, the exceptionally dense and regularized bilateral relationship between France and Germany provides both countries with opportunities to shape EU politics. As the two largest member states, France and Germany regularly set the EU’s political agenda. Via bilateral compromises that are acceptable for other member states, France-Germany might also initiate broader EU-level action (Krotz and Schild 2013). French-German bilateralism allows both countries to exercise European influence *together*. In addition, it might help each country *individually* to implement national preferences at the EU level via ‘pre-cooking’ and the support of the bilateral arena. Finally, Brexit, that is the United Kingdom’s withdrawal from EU membership, not only has increased the relative visibility of France and Germany as the two remaining ‘big’ member states. In addition, Brexit led the two countries to re-define and stress their responsibility for the survival and development of the EU even in an area like defense where historically, French-British relations had prevailed (Seidendorf 2022; Smith 2022: 27).

Thus, while the three levels of government might overlap in practice, for analytical purposes we consider them as distinct categories. Following the Europeanization literature (Börzel and Panke 2019), political influence-making can take the form of ‘top-down’ traveling, with processes at the European or bilateral level having consequences for the domestic arena. Alternatively, the ‘bottom-up’ perspective suggests that political events at the domestic or bilateral level shape decision-making in the European arena. In this study, we focus on the bottom-up perspective because we are interested in the way that French political priorities, Macron’s reform proposals, and a new balance within the French-German relationship shaped EU politics. In the following paragraphs, we demonstrate the three levels of government and their corresponding explanatory factors in more detail.

At the domestic level, during his first term in office (2017–2022), President Macron enjoyed a large room for political maneuver, especially with respect to French initiatives for European policymaking and reform. In the first place, this is due to the French presidential system, which gives the head of State an extent of authority and competences far exceeding those in other European political systems (Lequesne 1993; Rozenberg 2020). The French President is commander in chief of the armed forces, can push through legislative acts (with the help of the prime minister) without a vote in parliament, and represents the country in the European Council of national heads of State or Government. Due to the centralized French political and administrative system, and the pronounced role of the President of the Republic, Macron had a media and public attention unrivaled among other national leaders.

Several factors, which were specific to the 2017–2022 period, led the French system to fully enhance the President’s visibility and authority in European affairs. First, Macron’s political party, ‘La République en Marche’ (LREM), together with its allies, held an absolute majority in parliament. This secured political stability and the smooth adoption of legislation, at least with respect to EU politics. Second, Macron sought to “establish the European issue as the main cleavage for French politics” (Rozenberg 2020: 78–83). By contrast, the center-right and center-left parties, which had dominated French political life throughout the postwar years, now had significant Eurosceptic minorities in their ranks. This led them to adopt a double discourse combining pro-European and Eurosceptic elements. The 2017 French presidential elections arguably were the first European election where the “transnational cleavage” fully came to the fore (Hooghe and Marks 2018). Aggregating pro-EU voters from both the Right and the Left, LREM’s electoral basis provided Macron with an integrationist European mandate. Third, Macron’s victory and France’s ‘choice for Europe’ gave the new President some ex-ante credit and leverage among other national leaders (Bertoncini and Chopin 2020). In view of a possible Presidency of the far-right candidate, Marine Le Pen, many policymakers were relieved about the election outcome. They also recognized that the new President would need some successes to strengthen France’s pro-European electorate.

At the bilateral level, France and Germany have established a relationship that rests on close administrative and political ties which are unique even in the highly institutionalized environment inside the EU (Krotz and Schild 2013). Due to their size and large political, material and diplomatic resources, France and Germany, especially when acting together, are of a “critical mass” and at various occasions have decisively shaped the European integration process (Gruber 2000). French-German bilateralism and bargaining clout also provide both countries with opportunities to promote national priorities. Some scholars have noted an asymmetry in this bilateral relationship in favor of Germany (Bulmer and Paterson 2019; Webber 2019). Such an assessment, however, is usually based on the Euro crisis from 2009 to 2015, where German economic and financial weight came to the fore. By contrast, Germany’s leverage in the EU became more fragile in the context of the 2015/16 migration crisis. Finally, the lengthy government formation following the 2017 parliamentary elections, together with Chancellor Merkel’s announcement that this would be her last term in office, made German European initiatives unlikely (Lequesne and Schild 2018).

These developments and constraints on the part of Germany provided President Macron with opportunities to move France center stage again and put the bilateral relationship into a new balance. German leverage and guidance in the EU were in retreat precisely at the time when Macron came to power. The new French President knew how to use this constellation. He embarked on a discursive strategy addressing his European reform proposals explicitly toward Germany (Lequesne and Behal 2019). By doing so, he appealed to Germany’s European commitment, which was a call that the new government (that finally took office in March 2018) could no longer ignore. Next to several bilateral initiatives on European topics over the following months (see below), another result of Macron’s rhetorical strategy was a new French-German Treaty of Friendship signed in Aachen in January 2019 (France Diplomacy 2019). Therein, the two countries put their bilateral relation on a new basis and suggested EU-level action in policy fields where little change had happened in previous years, such as defense.

At the European level, after 2017 the EU and its member states faced several exogenous shocks calling into question certainties and principles which had long been taken for granted. A more volatile US foreign policy, notably with respect to European security and NATO; a more assertive China in global trade issues; and a belligerent Russia threatened the post-Second World War European security order. Such developments also challenged established European economic models that were based on multilateralism, global trade, and the cheap import of (energy) resources. In particular, the COVID-19 pandemic, which fully hit Europe starting from February 2020, led to an unprecedented recession of the European economy, reintroduced border closures, and the interruption of supply chains into and inside the EU’s single market. Such crises call on policymakers to provide fast responses to pressing problems against the background of great uncertainty. When they reveal shortcomings in existing structures and policies, they can be the driver for institutional and policy change (Boin et al. 2017).

Scholars have argued that “symmetric” crises affecting all member states to a similar extent are more likely to trigger change than “asymmetric” crises, which affect only a few actors while most member states are hardly concerned and/or have more promising national alternatives available compared to European action (Ferrara and Kriesi 2022). While France and President Macron suggested EU-level reforms and innovations across a range of policy fields, Germany has been considered a “status-quo power” which often seeks to preserve current structures and provisions (Schimmelfennig 2021). Thus, the sequence of several symmetric crises, such as the Covid-19 pandemic, threatening and undermining existing structures were likely to favor actors that advocated change, like France.

In the empirical part, we show how factors located at these three levels of government came together to strengthen France’s role in key EU policy fields and thus in EU politics more generally. We argue that the enhanced influence was due to the interplay of longer-term trends and punctuated shocks. On the one hand, in line with traditional French European priorities, Macron suggested a number of reforms in selected EU policy fields. The French government also developed the respective instruments and strategies, seeking to win over other national governments, primarily Germany. On the other hand, moments of crisis and uncertainty moved French-inspired proposals center stage and facilitated their implementation at the EU level.

To be sure, we do not argue that all the change we observe happened similarly rapidly and at the same time. Neither do we hold that the degree of change was equally dramatic in all policy fields. However, the literature on historical institutionalism and critical junctures (Capoccia and Kelemen 2007) leads us to consider and combine longer-term developments and short-term periods of crisis. Moments of uncertainty and high dynamics tend to favor actors with the necessary (administrative, material, etc.) resources and political will to promote change. Moreover, the new path taken is likely to draw from already developed and available institutional and policy instruments (see also Capoccia 2015: 151).

Admittedly, our suggested model is more complex than most scholarly accounts and the usual factors put forward to explain change and developments in the EU, such as national bargaining power, institutional rules, and the role of specific ideas. However, bargaining power between member states remained relatively stable during our period of interest. Similarly, the prevailing institutional rules in key decision-making arenas, such as the European Council, did not change. Finally, French European ideas and policy preferences have been remarkably stable over time, but in the past had not made an impact like they did during the first Macron Presidency. Thus, the longer-term preparation of EU instruments and measures at both the domestic and the bilateral level, together with punctuated shocks at the European level and the political agency of President Macron, provide a more complete analytical framework. Eventually, when crises forced policymakers to re-consider EU policy objectives and the way to achieve them, French priorities and targeted strategies to make them acceptable for other member states enabled Macron to decisively shape European politics and the EU’s political direction.

### Methods, data, and empirical procedure

To assess and demonstrate France’s clout on EU politics and how the theoretical model plays out in practice, we trace developments in three key EU policy fields during President Macron’s first term in office (2017–2022). This article seeks to identify and test explanatory factors for France’s (increasing) influence on EU politics. Accounting for the extent of French influence across *all* EU policy fields, however, would go beyond the scope of this article. We thus focus on fiscal policy, competition policy, and defense industrial policy, which are policy fields where significant change happened recently. In Gerring’s (2017: 56–58, 105–114) terminology, our cases are “typical” or “pathway” cases that demonstrate assumed causal relationships between theorized explanatory factors and observable outcomes.

In all three EU policy fields, French governments in the past repeatedly had tried to upload traditional and rather constant French preferences, but they only had been partially successful, if at all. Beyond the similarity of having experienced significant change in recent years, our cases show some variation: first, fiscal policy is a field where ‘Northern’ member states like Germany are usually considered to determine the political agenda. Second, competition law is a supranational policy field where the European Commission exercises considerable authority. Defense policy, finally, is considered a “core state power” where member states have been reluctant to pool competences at the European level (Genschel and Jachtenfuchs 2013). One would thus not expect ‘easy’ change in any of these policy field. If, however, significant change happened along French priorities and due to French strategies, one can infer some conclusions about France’s more general influence on EU politics and the EU’s overall political orientation.

Our analysis rests on a careful tracing of events and trends over the period from Macron’s election victory in May 2017 until the French presidential elections in April 2022. We build on a range of primary sources including 20 expert interviews with policymakers, civil servants, and think-tankers from France, other member states, and EU institutions. We pursued a semi-directed interview methodology, which combines both general and policy-specific questions (Cohen 1999: 6–7). Next, we consulted EU and national policy documents, European Council Conclusions, strategy papers, and opinion pieces. Aware of the methodological risks when focusing on primary sources, and on elite interviews in particular, we triangulate our data with secondary academic literature on the respective topics.

In the ensuing empirical part, we consider the three policy fields in turn. In each case study, we first provide background information on the historical context and traditional French preferences. Next, we trace events and developments at the domestic, bilateral, and European level. Together, the values that the explanatory factors take, and their interplay, determine the change in EU politics that we observe. We conclude each case study with an assessment of the most important outcomes and the EU’s outlook.

### Growing French influence over time and across EU policy fields

#### Fiscal policy

Since the inception of European monetary cooperation in the 1970s, French governments of both the political Left and Right persistently sought to establish an EU-level fiscal policy, albeit, at first, in an intergovernmental rather than supranational format (Howarth 2007). As a country with a traditionally current account deficit and pressures to devalue its currency, France, before the introduction of the single currency, advocated for common macroeconomic stabilization mechanisms. Such mechanisms promised France to obtain more “symmetric” adjustment costs and burden-sharing between surplus and deficit countries (Howarth and Schild 2017). During the Euro crisis from 2009 to 2015, French governments pushed for the introduction of Euro bonds, that is the joint issuance of government debt by member states. In doing so, French governments led a camp of other ‘Southern’ countries with similar fiscal priorities. When the Covid-19 pandemic hit Europe in early 2020, France again made the argument for joint fiscal efforts and instruments to fight the historic recession.

A central point in Macron’s 2017 Sorbonne speech was his proposal for “a stronger budget within Europe, at the heart of the Eurozone”. He considered this tool as the cornerstone of an “integrated Europe”. The purpose of the Eurozone budget was to finance common infrastructure projects, accelerate the digital and ecological transitions, and “provide stability in the face of economic shocks”. It was to be financed via “European taxes”, notably in the digital and environmental fields (Macron 2017). In the following years, in line with the country’s long-term fiscal preferences, Macron and his ministers insisted on new common budgetary instruments with a macroeconomic stabilization function to balance economic downturns. While the French administration was developing respective proposals, Macron sought to convince other national heads of State or Government, in particular the German Chancellor, of the need for such a budgetary instrument.

Germany, by contrast, due to moral hazard concerns and emphasis on national responsibility, traditionally opposes more centralization in European fiscal policy and greater risk-sharing among member states. Only in moments of high pressure and interdependence, and when status quo costs have become unbearably high, did Germany give up its resistance (Schild 2020). At the same time, however, research also has shown that in view of the privileged French-German relationship, and when confronted with a stark choice between France and other ‘Northern’ member states, Germany regularly sought compromises with the former. From a German perspective, the imperative of bilateralism and greater concerns about the EU’s cohesion and stability outweigh short-term calculations of fiscal preferences (Howarth and Schild 2022). In the following paragraphs, we show how French plans and preparations in EU fiscal policy first led to tentative initiatives at the bilateral level together with Germany. A moment of deep crisis then opened a window of opportunity to realize and go beyond these plans on a much larger scale at the European level.

Macron’s proposal in his Sorbonne speech for a Eurozone budget worth several points of Eurozone GDP met the expected criticism of ‘Northern’ member states opposing the idea of greater common fiscal resources. At a first glance, Germany, caught in lengthy coalition talks, did not give an answer to Macron’s calls. Behind the scenes, however, pressures increased to take a stance. Several policymakers from their own parliamentary groups pushed the new government, which took office in March 2018, to send a European signal (Interviews 2, 5). The coalition agreement, captioning “A new departure for Europe”, referred to “Europe” no less than 298 times (deutschland.de 2018). Three months later, in their Meseberg Declaration of June 2018, France and Germany proposed establishing “a Eurozone budget within the framework of the European Union”, to be started in 2021 and financed from national contributions, tax revenues, and “European resources” (Meseberg Declaration 2018). On the insistence of the German government, no concrete volume was indicated. Germany also attached more importance to “competitiveness and convergence” than to “stabilization” and the redistributive “transfer” dimension. However, what mattered from the French point of view was that Germany, for the first time, had subscribed to the idea of a standalone budgetary instrument, next to the EU’s regular Multi-annual Financial Framework (MFF). This not only marked a “momentous shift” in the German position, but it also altered political dynamics in Europe (Enderlein and Guttenberg 2018).

At the European level, several Northern member states, which grouped as the ‘New Hanseatic League’, sought to water down the French-German proposals (Schoeller 2021). In June and October 2019, Eurozone finance ministers agreed on the features of the Eurozone budget. Formally labeled the ‘Budgetary Instrument for Convergence and Competitiveness’, it was to have a volume of a mere €30 billion with little redistributive purposes. However, unexpected events altered the course of action. Due to the onset of the Covid-19 pandemic in Europe in early 2020, the BICC was never implemented. Instead, the pandemic crisis, which triggered the largest recession in the EU’s history, made more comprehensive measures necessary.

At first, France and Germany found themselves at opposite ends of member states’ stances on the crisis: while France advocated bold European fiscal measures and support for the hardest hit countries, Germany suggested using existing instruments. On 25 March 2020, Macron, together with eight other national leaders, signed a letter to the European Council President calling for joint borrowing (Euractiv 2020). Over the following weeks, in the face of French pressure, rising infection numbers, and the economic downturn, the German government reconsidered its position (Krotz and Schramm 2022). On 18 May, President Macron and Chancellor Merkel advocated a European “reconstruction fund” worth €500 billion. It was to be financed via common borrowing and entirely based on grants for the countries hardest hit by the pandemic (France Diplomacy 2020). About one week later, the Commission issued a proposal for a European recovery instrument, termed ‘Next Generation EU’ (NGEU). To the €500 billion in grants, as suggested by France-Germany, the Commission added another €250 billion in loans (European Commission 2020). According to the plan, the Commission was to be allowed, backed by member-state guarantees and within the framework of the MFF, to raise debts in the financial markets.

EU and national civil servants confirm that in its purpose and governance structures, NGEU to a large extent builds on the BICC (Interviews 3, 4). Both instruments constitute a budgetary tool that is complementary to the regular MFF. At the insistence of Northern countries, NGEU, for the time being, is a temporary instrument that is supposed to expire in 2026. Its debts, however, will have to be paid back until 2058. Moreover, with its volume of €750 billion, NGEU comes close to the stabilization function that Macron had called for. For several member states, especially in Europe’s South and East, the allocated grants and loans represent numerous percentage points of their GDP and thus can be considered macroeconomically relevant. Finally, NGEU is a redistributive instrument in that those member states that had already been in a weak fiscal position before the pandemic benefit the most. In Macron’s words, NGEU enables “real financial transfers” between member states (Bundeskanzlerin 2020).

With the recovery plan adopted, and to counter the EU’s most recent challenges, French policymakers soon called to turn NGEU into a permanent instrument. Others suggested the creation of a similar fiscal instrument. Indeed, in view of Russia’s war against Ukraine, energy shortages, and rising inflation in Europe, such proposals started gaining ground within EU institutions and member states. In March 2022, the Commission established the ‘Repower EU’ instrument to reduce Europe’s dependence on Russian fossil fuels and fasten its ecological transition (European Commission 2022b). To mobilize the necessary fiscal means, the Commission suggested using the Recovery and Resilience Facility (RRF), which forms the centerpiece of NGEU. Like the BICC before, the RRF might become another instance of provisions and instruments, once established at the European level, being used and developed further for new purposes. Moreover, the need to pay for the RRF has reinvigorated the idea of collecting EU-level fiscal resources and opened the way for the agreement on a carbon border adjustment mechanism in March 2022 (Interview 17).

#### Competition policy

Since the earliest decades of European integration, successive French governments have been skeptical toward EU competition rules on the grounds that they undermine industrial policy instruments including state aid and the government-supported creation of “European champions” (Warlouzet 2019). First, they sought lifting restrictions on state aid in order to allow governments to allocate resources to industries they consider strategic (Clift 2013). Second, French governments have long called the European Commission to revise the criteria it uses for merger control. Rather than looking at a newly formed company’s market dominance at the national or European level, Commission officials were encouraged to think in terms of global competition (French Senate 2007). A 2019 report on competition policy requested by the French ministry of the economy clearly shows that both policy objectives are very much alive (Inspection Générale des Finances 2019). Policymakers link competition policy reform to what appears as France’s longstanding affinity for industrial policy. Questioned on the matter, an official from the French Permanent Representation to the EU exclaimed: “of course we have always advocated for EU industrial policy, we are French!” (Interview 8) Similarly, an official from a Northern European member state pejoratively remarked that “with the French, industrial policy is a mindset” (Interview 19). In sum, successive French governments’ ambition to “export their model of industrial intervention to the European level” is a longstanding preference (Cohen 1992: 22). While French preferences in competition policy are very old, they were fairly isolated within the EU until recently. EU competition rules and the Commission’s Directorate-General tasked with implementing them, DG COMP, historically had been dominated by a market-oriented policy program (Smith 2022: 16–19).

Even before his election as President, Macron re-oriented his predecessors’ competition policy reform agenda. As minister of the economy and aspiring presidential candidate, he stated that “competition policy cannot be exclusively intra-European” given that both “the Chinese and Americans create global giants.” Accordingly, EU competition policy had “lost the terms of sovereignty” and needed to incorporate recent developments (Macron 2016). Following his election as President, Macron used his newly acquired EU-level political clout to pursue these priorities. In his 2017 Sorbonne speech, he linked “European sovereignty” to the creation of “European champions” in technology-intensive industries (Macron 2017). While Macron’s visibility on the European scene reinvigorated French ideas about competition policy reform, they were also divisive. This became apparent in the Alstom-Siemens case which envisioned the merger of the largest French and German train manufacturers to create a ‘European champion’. French officials justified this merger on the grounds of competition from the Chinese railway manufacturer CRRC, which is by far the world’s largest player in its sector (Politico 2018). In stark contrast, competition authorities from the UK, the Netherlands, Belgium, and Spain sent a joint letter of caution to the Commission warning about the excessive concentration that this merger would entail (Coscelli et al. 2018). Furthermore, an official from DG COMP noted that there was a “total misunderstanding between France and Brussels” given the Commission’s suspicions that “when France speaks of a European champion, they really mean a French champion” (Interview 18).

The Commission’s decision to refuse the Alstom-Siemens merger in February 2019 initially suggested that French preferences would continue to be isolated in EU competition policy. However, the Alstom-Siemens case ultimately acted as the catalyzer for a broader reform of competition rules. A French official remarked that “Alstom-Siemens has pushed Germany to [the French] side” (Interview 6). Just weeks after the Commission’s decision, the French and German governments published a joint manifesto for an “industrial policy fit for the twenty-first century” where they envisioned a comprehensive set of competition policy reforms including an update of merger guidelines and even to give a “right of appeal to the European Council to override Commission decisions” (Altmaier and Le Maire 2019). Given Germany’s historical commitment to stringent competition rules following its ordoliberal doctrine, this was a major shift in preferences and a decisive development for the EU (Warlouzet 2019). As an official from DG COMP put it: Germany has been the "swing state" between liberal and interventionist countries (Interview 11). In addition to broader changes in the global political economy, French-German bilateralism allowed for other factors to come into play: The controversy surrounding the publication of the February 2019 industrial strategy paper indicated that the German minister of the economy, Peter Altmaier, did not receive unanimous support, neither within his own ministry nor from business groups. However, characterized by different observers as a “Francophile”, an “old friend” of his French counterpart, Bruno Le Maire, and “personally invested in the Alstom-Siemens deal”, Altmaier turned out to be a crucial ally for France (Interviews 1, 12, 19).

In December 2019, the Commissioner for Competition, Margrethe Vestager, gave the first signals that the EU was considering an update of its merger control guidelines (Vestager 2019). This change finally materialized in July 2021 as the Commission updated its market definition criteria for the first time since 1997 (Meunier and Mickus 2020: 1085). Especially relevant with respect to the Alstom-Siemens case is that the Commission started considering “conditions of globalization and import competition” from non-EU producers (European Commission 2021). The update of merger guidelines was only one prominent success in competition policy that France obtained during the Macron Presidency. Another proposal mentioned in the French-German manifesto for industrial policy had been to make greater use of the “recent novelties in European state-aids rules” and the framework of Important Projects of Common European Interest (ICPEI). While article 107.3 of the 1992 Maastricht Treaty briefly mentions “important projects of common European interest” where governments can obtain a derogation from the Commission to provide state aid, no IPCEI came into being in the first 26 years following the introduction of this Treaty. In stark contrast, five different IPCEIs have been approved by the Commission in the period thereafter. In total, more than €18 billion^1^ of government funding were allocated to three different industries in less than four years, while far greater amounts of private investment are expected to be lured in. From a previously obscure sub-article of the Maastricht Treaty, IPCEIs thus became a full-fledged concept in EU law. In the following years, the Commission approved projects in microelectronics (2018), electric car batteries (2019, 2020), and hydrogen fuels (two in 2022). The French and German governments took the lead, financing more than half of the total project expenses.^2^

Although the first French-German initiatives to change state aid rules and codify IPCEI date from the mid-2010s, the succession of recent crises hitting the EU and disrupting global supply chains accelerated these developments (Interview 7). Most remarkably, the Covid-19 pandemic led the European Council (2020) to “invite the Commission to identify strategic dependencies, particularly in the most sensitive industrial ecosystems such as for health, and to propose measures to reduce these dependencies, including by diversifying production and supply chains, ensuring strategic stockpiling, as well as fostering production and investment in Europe”. In the following two years, the European Commission, and particularly the French Commissioner for the internal market, Thierry Breton, became actively involved in industrial policy (Interview 15). The Chips Act proposal from February 2022 foresees a further €30 billion of public investment into semi-conductor foundries over an eight-year period and is similarly expected to bring changes to EU state aid rules. A new regulation justifies these government subsidies on the grounds that they are allocated to a factory that is “first-of-a-kind” in the EU on the condition that member states have priority access to its production output. Much like IPCEIs, the first-of-a-kind exception had existed for decades, but it was only recently activated (European Commission 2022a; Interview 14). In sum, previous French-German cooperation and activism, together with intensifying challenges of globalization, served as a critical juncture allowing the French government to realize its longstanding ambition to reform EU competition policy.

#### Defense industrial policy

In recent years, some observers argued that President Macron and the French government failed to impose their vision of “European strategic autonomy” (Taylor 2022) and that Macron’s concrete proposals in defense policy did not match the ambition of this idea (Vallée 2022). For instance, the European Intervention Initiative is an ad hoc and intergovernmental instrument which is situated outside the EU’s institutions. Furthermore, both “strategic autonomy” and “European sover eignty” remain contentious when it comes to operational aspects of defence policy. A senior European diplomat reports that “even French diplomats admit that European sovereignty does not exist and that it’s a political term” (Interview 13). However, with respect to defense policy, it is important to note that Macron’s actual objectives concern defense *industrial* policy. An official from the Commission’s Directorate-General for defense industry, DG DEFIS, explained that “strategic autonomy is a French priority because around half of the EU’s defense industries are French” (Interview 18). Indeed, the French government essentially wants to create a protected defense market at the EU level to exclude third-country producers and ensure the competitive advantage of its own companies (Calcara and Simon 2021).

Béraud-Sudreau and Pannier (2021: 300–301) remark that “strategic autonomy was a priority of France’s European policy since the 1990s” and that the country “pushed this term to become part of the European vocabulary.” A longstanding component of the strategic autonomy agenda, still intact under the Macron Presidency, has been the emergence of companies of critical size that are able to compete with US producers (Faure 2020a: 93). Arguably, “strategic autonomy is not primarily about defense itself but the mastery of critical military technologies” (Interview 10). In the past, France’s ability to take EU-level defense industrial policy initiatives had often been jeopardized by internal divisions preventing it from forming a unitary national position. While the country’s larger defense firms and officials from the economy ministry sought to create a Europe-wide defense market and sided with the Commission, officials from the defense ministry and from the national champion, Dassault Aviation, were more wary. As a result, French state institutions often held competing policy positions, for instance during the negotiations of the 2009 “defense package” (Faure 2020b).

To be sure, defense industrial policy remained divisive during Macron’s Presidency. This notably became evident in the difficult negotiations with Germany on FCAS, the Future Combat Air System (Defense News 2022). As previously noted, Macron’s stance on European sovereignty does not lack its own ambiguities when it comes to defense policy. Nevertheless, the French President was consistent in his support for the creation of a supranationally governed European Defence Technological and Industrial Base (Faure 2020b: 15–17, 19–21). Having the support of many EU-level stakeholders, a defense industry lobbyist explained that “[they] hoped him to stay in power” (Interview 9). Leaked emails further revealed that Macron and his party had already developed a working relationship with the Commission on defense industrial policy during his first presidential campaign (Béraud-Sudreau and Pannier 2021: 300).

Moreover, Germany’s defense industrial policy preferences already converged significantly with Macron’s agenda, as the country traditionally favors a “process of positive state-building in military force generation” (Biermann and Weiss 2021: 228). The large size of their defense market also meant that German policymakers shared France’s preference for “high-entry barriers” to non-European producers (Calcara and Simon 2021: 864). Following the French-German Security Council in July 2017, merely two months after Macron’s election, the two governments announced a comprehensive bilateral cooperation agenda for defense industrial policy including the joint development of two of the costliest armaments programs in European history: the Main Ground Combat System (MGCS) and the FCAS (Kempin and Kunz 2017).

Similar to the other two policy fields, EU defense industrial cooperation had already gained some momentum during the mid-2010s as a network of French and German government officials, together with Commission civil servants, made creative “usage” of international crises, such as Brexit, the Russian annexation of Crimea, and the US’s orientation toward Asia, to justify European integration (Béraud-Sudreau and Pannier 2021). This “configuration” of defense industry stakeholders thus long preceded the Macron Presidency (Faure 2020a; 2020b). Yet again, Macron’s election and subsequent role in articulating the ‘European sovereignty’ discourse allowed France to become a much more critical node in this network, especially after the appointment of Thierry Breton as internal market commissioner in 2019.

While it still seems premature to make any final assessment about the effects of the two French-German programs MGCS and FCAS on EU defense industrial integration, the most prominent development during the time covered in this study has been the establishment of the European Defence Fund in 2017. This very first instance of EU-level defense spending will amount to €8 billion^3^ in the 2021–2027 period, making the EDF the third largest defense R&D budget in the EU after those of France and Germany. While the EDF officially came as a Commission initiative, Calcara and Simon (2021: 875) remark that “president Emmanuel Macron’s impulse and cooperation with Commission President Jean-Claude Juncker provided the political foundation for the EDF and ensured that the initiative kept momentum and support”.

As other member states feared an intergovernmental format enabling France to assume control over the program, accepting a prominent role for the Commission was inevitable (Haroche 2020: 864). Supranational decision-making did not leave the French government out of the loop, however. DG DEFIS, the Commission’s newly created Directorate-General tasked with the implementation of the EDF, was placed within the portfolio of the Commissioner for the internal market. France had successfully obtained this portfolio during EU-level negotiations in 2019 and nominated Sylvie Goulard, the country’s defense minister. Following the European Parliament’s rejection of her nomination, the French government designated Thierry Breton. Having previously been France’s minister for the economy (2005–07) and CEO of cybersecurity company Atos, Breton became Macron’s close ally in the Commission. In the words of an EU diplomat, “other member states often suspect that there is a direct line between Thierry Breton and the Élysée Palace” (Interview 19). Furthermore, allocating funds on a competitive basis rather than through the ‘juste retour’ principle is highly beneficial to France’s defense sector, the largest and most competitive among EU countries. French producers consequently led the first year of EDF funding, as was announced in July 2022 (La Tribune 2022). Critically, access to third-country entities has been restricted by article 9 of the regulation establishing the EDF (Official Journal 2021). In this regard, France’s negotiation position was helped by the fact that the Commission grounded the EDF proposal on a legal basis of industrial policy rather than defense (Martins and Mawdsley 2021). A DG DEFIS official explained that otherwise, “if we bought non-European products, there would not have been a legal basis for the Commission to act” (Interview 18).

In May 2022, following Russia’s war against Ukraine and the threat to Europe’s security order, the Commission proposed a joint weapons procurement regulation. Although limited to €500 million, EU-level armament purchases are unprecedented, and the regulation may be turned into a permanent instrument in the future. Like the EDF, the regulation excludes third-country entities (European Commission 2022a, art. 8). Once more, a geopolitical crisis had benefited French priorities, this time in defense industrial policy (Interview 20). According to one official, “after hostilities had started, [French authorities] quickly noticed that the war would reinforce the pertinence of the French EU presidency’s agenda of strategic autonomy” (Interview 16).

## Conclusions

“[T]he time when France makes proposals in order to move forward with Europe and every European who so wishes—that time has returned.” With these words, President Macron finished his Sorbonne speech in September 2017. He described this strategy as the third, most adequate and most promising path compared to the alternatives: on the one hand, the path of France taking decisions for Europe never really existed and remains unlikely in a Union of (formally) equal member states. On the other hand, France taking decisions irrespective of what other member states were doing, was not an option either (Macron 2017).

In 2017, few observers would have imagined the prospects of success of Macron’s proposals. However, as this article has explained, in recent years France played a remarkable and, in many respects, predominant role in EU politics. Other than many scholarly accounts suggest, during his first term, President Macron, together with his government, managed to lead other member states and EU institutions to implement traditional French preferences and priorities. Thus, France’s influence on specific EU policies and on the EU’s overall political direction also is larger than many policymakers acknowledge, not least in France.

We have argued and shown that this clout is due to several explanatory factors, located at different levels of government, coming together. First, a benevolent domestic environment characterized by a centralized political system, a solid majority in parliament, and a pro-European government enabled Macron to propose and pursue bold European initiatives. Second, a self-confident French President with a clear agenda and concrete proposals, together with a German partner caught in domestic difficulties and without European initiatives, shifted the balance within the bilateral relation toward France. Third, several external and largely symmetric shocks hitting the EU, such as the pandemic, global economic competition, volatile transatlantic security ties, and warfare in Europe’s neighborhood, favored the reform-oriented French European political agenda.

These explanatory factors and their specific interplay explain France’s influence in the EU, at least in the three policy fields analyzed in this study. Next to the necessary economic and financial resources, diplomatic and administrative capacities, and political authority, the influence that a (large) member state exercises in the EU thus follows certain waves defined by important background conditions and events. In the same way as Germany’s supposed dominance in the EU, most notably during the Euro crisis, was context and policy-field specific, so did France profit from a number of exogenous crises. A German decade in the EU (2007–2017) might thus be followed by a French decade. At the same time, and irrespective of such contingency, this article demonstrates that political consistency, persistency, and strategy do pay off. We have shown how French political elites, sometimes over decades, had developed and prepared European initiatives before, under President Macron, they eventually managed to realize them.

Two clarifications and two qualifications must be made. In terms of clarifications, Macron’s proposals and initiatives first and foremost were intended to advance France’s interests. French European politics remains national interest politics. This is neither surprising nor exceptional, since all member states promote national interests in and via the EU (Chopin and Faure 2022; Parsons 2003). However, in view of Macron’s many ‘pro-European’ discourses, this point deserves to be stressed. A key manifestation of France under Macron seeking to organize EU politics according to French interests has been that Macron selectively favored either supranational or intergovernmental patterns: in fiscal policy, France proposed supranational methods like Commission borrowing. In defense policy, by contrast, not least in an attempt to protect France’s defense industry, Macron at times insisted on intergovernmental European decision-making procedures (Faure 2020c).

Second, it was not only Macron who suggested certain policy instruments which eventually were implemented at the European level. Other member states like Italy, for instance, for a long time had advocated for a Eurozone budget. However, over time and across policy fields, it was France that decisively promoted and realized European policy proposals. France simply mattered more than other member states. A key explanatory factor here is French-German bilateralism. Via this institutionalized and routinized relationship, both countries, together but also individually, shape EU politics. Appealing to Germany’s European commitment, Macron managed to put the relationship on a more equal footing. He also used this strategic tool to have the German government agreeing on several bilateral compromises, which could later be expanded to the European level.

With respect to qualifications, the June 2022 legislative elections made Macron’s political life more difficult. The re-elected President lost ‘his’ parliamentary majority and now faces strengthened groups on both the political Right and Left. Ironically, it appears, French voters reduced their President’s room for maneuver at the EU level at a time when his influence was greatest. Only the next years will tell how this reduced domestic room for maneuver, together with the fact that this term will be Macron’s last one in office, affects the President’s performance on the European stage. Similarly, recent substantial differences and open public clashes between French and German government representatives on EU energy and security policy (Kauffmann 2022) suggest that effective bilateralism relies on good interpersonal relations, primarily between the President and Chancellor. With a new officeholder in Berlin, French-German cooperation has become more unpredictable.

Finally, with respect to Russia’s war against Ukraine and the EU’s energy crisis, France faces considerable opposition from several member states, particularly in Central and Eastern Europe. As we have shown in this article, in recent years the EU has taken significant steps with respect to common security and defense. Nevertheless, most member states continue to favor a strong role of both the US and NATO. They are also suspicious about France’s historically close ties with Russia and Macron’s attempts to mediate with President Putin. This constellation of actors and conditions again highlights that several factors, located at different levels of government, together determine a (large) country’s influence in the EU. Overall, during Macron’s second term, France’s role and potential appear more uncertain due to domestic and broader European developments.

## Footnotes

1 The amount of €18 billion is an estimation compiling publicly available information on each project, namely €1.7 bn in electronics, €6.1 bn in batteries, and €10.6 bn in hydrogen.

2 Data is not publicly available on every project. This estimate is based on the microelectronics IPCEI (€1.2 bn French-German expenditure out of a total €1.75 bn) and the first of the two batteries IPCEI (€2 bn French-German expenditure out of a total €3.2 bn). In these two projects, French-German investment accounts for 64% of overall European investment.

3 Initially envisioned to be €13 bn, its amount was reduced in the context of the Covid-19 pandemic.
